# Toward Innovation in Healthcare: An Analysis of the Digital Behavior of Older People in Europe and Japan for the Introduction of a Technological Coaching System

**DOI:** 10.3390/healthcare12020143

**Published:** 2024-01-08

**Authors:** Johanna Möller, Vera Stara, Giulio Amabili, Federico Barbarossa, Giovanni Renato Riccardi, Clotilda Martella, Valentina Di Donna, Cecilia Palmier, Toshimi Ogawa, Marine Luc, Rainer Wieching, Elvira Maranesi, Roberta Bevilacqua

**Affiliations:** 1 Diözesan-Caritasverband für das Erzbistum Köln e.V., D-50676 Cologne, Germany; johanna.moeller@caritasnet.de; 2 Scientific Direction, IRCCS INRCA, 60124 Ancona, Italy; g.amabili@inrca.it (G.A.); f.barbarossa@inrca.it (F.B.); r.bevilacqua@inrca.it (R.B.); 3 Clinical Unit of Physical Rehabilitation, IRCCS INRCA, 60121 Ancona, Italy; g.riccardi@inrca.it; 4 Clinical Unit of Nephrology and Dialysis, IRCCS INRCA, 60121 Ancona, Italy; c.martella@inrca.it; 5 Clinical Unit of Physical Rehabilitation, IRCCS INRCA, 63900 Fermo, Italy; v.didonna@inrca.it; 6 Service de Gériatrie 1&2, AP-HP, Hôpital Broca, 75013 Paris, France; cecilia.palmier@aphp.fr; 7 Smart-Aging Research Center, Tohoku University, Sendai 980-8575, Miyagi, Japan; toshimi.ogawa.e6@tohoku.ac.jp; 8 AGE Platform Europe, 1150 Brussels, Belgium; marine.luc@age-platform.eu; 9 Institute for New Media and Information Systems, University Siegen, D-57072 Siegen, Germany; rainer.wieching@uni-siegen.de

**Keywords:** older people, ICT for aging well, socioeconomic assessment, multidisciplinary study, impact of technology, gerontechnology, acceptance, attitude toward technology, active and healthy aging, digital device

## Abstract

(1) Background: The increasing older population and demographic shifts highlight the need to understand the digital profiles of older adults, a pivotal factor in developing innovative technologies like the e-VITA virtual coach. This personalized coach provides recommendations for sustainable well-being in a smart home environment. (2) Methods: This study focuses on analyzing the characteristics of older individuals categorized as Internet users (onliners) and non-users (offliners). European Social Survey data from 2021 were utilized for European analysis, determining Internet usage based on frequency. Offliners are defined as users who never use the Internet, and onliners as those who use it, albeit with different frequencies. In Japan, data from the 9th International Comparative Survey on the Lives and Attitudes of the Elderly were employed, based on the responses of 1367 subjects, which defined onliners as individuals using communication devices and offliners as those not utilizing fax machines, cell phones, or the Internet. (3) Results: This paper presents a primary analysis of older end-user context and perspectives, outlining effective strategies for the diffusion of an active and healthy aging coaching system in the market and society. (4) Conclusions: the study emphasizes the importance of analyzing digital behavior in any user-centered design approach to ensure the system’s acceptance after deployment.

## 1. Introduction

Due to the increasing lifespan and demographic changes, the older adult demographic is growing as potential internet users [1], as the number of Internet users among individuals over 70 has risen from 29% in 2014 to 52% in 2020 in Germany. However, a significant gap persists when compared to the nearly 100% Internet usage among those aged 14 to 49 [2]. Similar patterns are observed in France, where 100% of 18- to 24-year-olds use the Internet, contrasting with 58% of those over 70 [3]. In Italy, the 2019 data reveal a substantial Internet usage gap between individuals aged 25–34 (85%) and those aged 65 and over (29%) [4]. Various reasons contribute to the non-use of the Internet. Helsper and Reisdorf [5] explored a British sample to identify prevalent reasons for both non-use (individuals who have never used the Internet) and discontinued use (ex-users who used the Internet in the past but no longer do). The primary reason for non-use, cited by 50%, was a lack of interest. Additionally, one-tenth of the respondents mentioned a lack of access, high costs, and a lack of skills. Among ex-users, lack of access and high costs were the predominant reasons for discontinuation. Digital divide research indicates that socioeconomic factors such as education, income, age, gender, and migration background play a significant role [6]. The use of the Internet is steadily expanding among older people in Japan [7]. According to the Ministry of Internal Affairs and Communications’ Survey of Telecommunications Usage Trends (2018), 75.7% of people in their 60s, 53.6% of people in their 70s, and 23.4% of people aged 80 and older use the Internet. Usage rates for all these age groups have risen from 2010 to 2016. In the prevalent environment of widespread Internet use, the ability to comprehend the Internet accurately and make independent information choices becomes crucial for maintaining health.

Understanding the digital profiles of older users is a pivotal step in designing new technologies, particularly to support the health and well-being of the vulnerable older population [8]. Numerous studies claim that in order to coach older individuals to remain active and independent while preserving their intrinsic capacity, it is important to use technology as the basis of multi-component interventions [9].

The European project e-VITA (grant agreement n. 101016453) focuses on empowering older adults via a socio-technological support system, the e-VITA virtual coach, in Europe and Japan. The multidisciplinary consortium collaborating in this project will develop an innovative ICT-based virtual coaching system to detect changes in physical, cognitive, psychological, and social domains of older adults’ daily lives. The e-VITA virtual coach will thus provide personalized recommendations and interventions for sustainable well-being in a smart living environment at home by incorporating virtual coaches (social robots), sensor systems, chatbots, a social platform, and a smartphone app. These components, together with a main software named Use Cases Configurator, constitute the Virtual Coach. The goal of this analysis is to understand older people’s digital behavior and profiles, differentiating between Internet users and non-users. Knowing which categories of older adults use the Internet and which do not is crucial in determining how e-VITA should be introduced to the target group in the upcoming months. Additionally, it helps identify specific groups of individuals that may require special consideration in the development of the virtual coach. Data from the European Social Survey (ESS) and the 9th International Comparative Survey on the Lives and Attitudes of the Elderly were utilized for Italy, France, Germany, and Japan. Socioeconomic, health, and psychological factors were examined separately for each country to gain insights into the introduction of e-VITA to the target group.

## 2. Materials and Methods

### 2.1. Methodology Applied in Europe

The European Social Survey (ESS) is a survey conducted regularly in over 35 countries to date (30 in the ninth round used here) [10]. Its objectives are to observe and interpret public attitudes and values in Europe, to further develop methods to better measure cross-national surveys, and to develop European social and attitudinal indicators. The survey involves a random sample with a target response rate of at least 70%. The survey period for the data used here spanned between 2018 and 2019 (France 10/19/18–01/04/19, Italy 12/17/18–10/03/19, and Germany 08/29/18–04/03/19) (European Social Survey, 2021). People aged 65 and older were included in the analysis. The analysis focuses on the characteristics of older people who use the Internet (onliner) and people who do not use the Internet (offliner). For this purpose, Internet usage was determined based on the following question: “*People can use the internet on different devices such as computers, tablets and smartphones. How often do you use the internet on these or any other devices, whether for work or personal use?*” and answer options were 1 (Never), 2 (Only occasionally), 3 (A few times a week), 4 (Most days), or 5 (Every day). This was dichotomized in the present analysis, and people with a value of 2–5 were defined as “onliner”, while people with a value of 1 (Never) were defined as “offliner”. In addition to the usage variable described, the duration of use in minutes was also considered. This is surveyed in the ESS with the following question: “*On a typical day, about how much time do you spend using the internet on a computer, tablet, smartphone or other device, whether for work or personal use?*”.

Differences between onliners and offliners over 65 years of age with respect to various socioeconomic and health-related outcomes were considered. Socioeconomic variables represent age, number of children (n), number of grandchildren (n), gender (female/male), education level (low/middle/high), and migration background (yes/no). Table 1 shows how the variables mentioned are composed and how they were included in the analysis.

The educational level is composed of the International Standard Classification of Education (ISCED), which was developed by UNESCO in the early 1970s to provide a uniform framework for the collection and presentation of education statistics (BMBF, n.d.). The ES-ISCED (European Survey—ISCED) is an adapted European survey version of ISCED. In addition to socioeconomic characteristics, health and psychological aspects are also taken into account. These include happiness, subjective health, trust in others, and participation in social activities compared to others of the same age. Table 2 shows the composition of the variables mentioned and how they were included in the analysis.

Since a median split, according to Lasarov and Hoffmann [11], can significantly affect the results, the cut-off value for dichotomizing the variables (Satisfaction with health; take part in social activities) was set based on the scale. In addition to the variables mentioned above, religiousness was also considered. This is surveyed in the ESS with the question “*Regardless of whether you belong to a particular religion, how religious would you say you are?*” and response options ranging from 0 “Not at all religious” to 10 “Very religious”.

### 2.2. Methodology Applied in Japan

The data for Japan were obtained from the results of the 9th International Comparative Survey on the Lives and Attitudes of the Elderly, conducted by the Cabinet Office in 2020–2021. This survey was conducted in four countries: Japan, the United States, Germany, and Sweden. The subjects were men and women aged 60 and over living in each country, excluding those in institutions. In Japan, a mail survey was conducted in January 2021 to 2500 people selected by stratified two-stage random sampling. Based on the responses of 1367 valid respondents on the use of communication devices, onliners and offliners of the Internet were defined as follows. Onliners in the Japanese survey were defined as those who used e-mail, cell phones, or smartphones, gathered information, or shopped on the Internet. Offliners in the Japanese survey were defined as those who did not use a fax machine, cell phone or smartphone, or the Internet.

## 3. Results

### 3.1. Analysis of the Results from European Countries’ Data

First, those excluded from the data set who were younger than 65 years at the time of the survey and did not live in Germany, Italy, or France. This was followed by a frequency analysis of the sociodemographic variables and the health-related and psychological aspects. This process was country-specific for the entire sample and took into account usage behavior (onliners vs. offliners). For metric variables, the mean value, as well as the standard deviation, was determined. The distribution provides an initial impression of the extent to which onliners and offliners differ in terms of socioeconomic, health-related, and psychological aspects. In addition, a chi-square test was calculated between usage behavior and all other categorical variables, as well as the effect size of Cramer’s V. For nominal and metrically scaled variables, the Mann–Whitney U test was applied due to the lack of normal distribution. In addition, the effect size r is determined, which is also composed of the z-score and the root of N (*r* = $\frac{z}{\sqrt{N}}$). Values > 0.3 can be considered a medium effect size, values > 0.5 a large effect size [12]. The significance level for all analyses was set at *p* ≤ 0.05. For all analyses, data were weighted and corrected for population size. The weighting factor (analysis weights) is already provided in the ESS.

**Figure 1:**
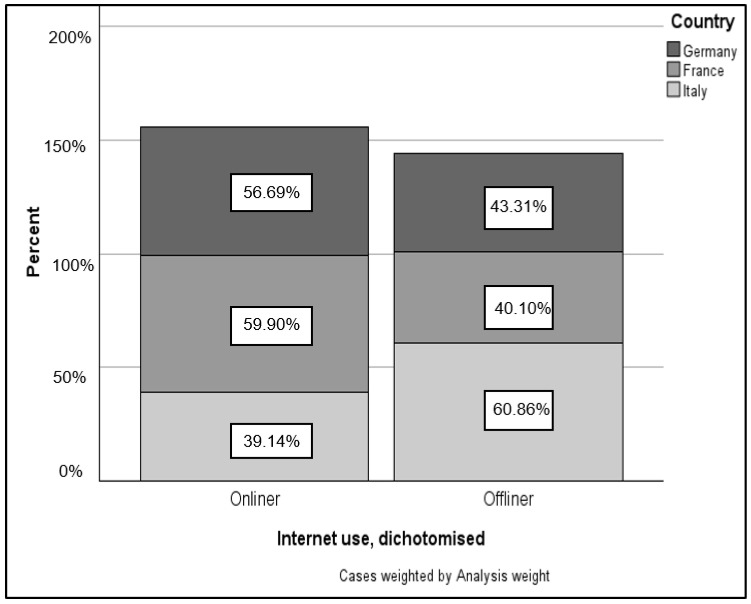
Proportion of onliner and offliner within countries.

A total of 1737 individuals could be included in the analysis for Germany, 1336 for France, and 1406 for Italy. First, it was examined how online and offline users are basically distributed within the countries. The results of the analysis are shown in Figure 1.

It shows that more than half of the 65-year-olds in France (59.90%) and Germany (56.69%) already use the Internet. In contrast, a different picture emerges from the Italian data. There, only 39.14% of the over-65s can be described as onliner, which means that more than half do not use the Internet. Except for Italy (n = 3), there were no missing values with regard to this analysis.

In terms of Internet usage in minutes, however, Italy leads with an average of 108.98 (±93.11) min, followed by Germany with an average of 102.89 (±83.72) min and France with an average of 8.43 (±91.84) min. However, the number of valid values is limited at this point. For the analysis of Internet use in minutes, 287 valid cases could be included for Italy, 549 for France, and 664 for Germany.

#### 3.1.1. Italy

Overall, the average age of the persons included in Italy is 74.89 (±7.00) years. The average number of children is about 2 ($\bar{X}$ = 2.12 ± 1.03), and the average number of grandchildren is above 2 ($\bar{X}$ = 2.48 ± 2.49). Onliners are, on average, 71.19 (±5.33) years old and have, on average, just under 2 children ($\bar{X}$ = 1.95 ± 0.86) and around 2 grandchildren ($\bar{X}$ = 1.89 ± 2.24). In contrast, offliners are significantly (*p* < 0.001) older on average at 77.27 (±6.91) years, have significantly (*p* < 0.001) more children ($\bar{X}$ = 2.21 ± 1.11), and, on average, have under 3 grandchildren ($\bar{X}$ = 2.84 ± 2.56), which is significantly (*p* < 0.001) less than onliners. There were no missing values with respect to the age variable. However, there were missing values for the number of children (n = 190) and grandchildren (n = 245). Table 3 shows the sociodemographic characteristics broken down by Internet use and for the Italian sample as a whole. It shows that aspects of low social status, such as female gender, low educational status, and the presence of a migration background, are found significantly more often among offliners than onliners (*p* < 0.001). They are also less likely to be married or in a partnership, more likely to be divorced, and more likely to be single (*p* < 0.001).

The largest effect sizes in the chi-square test are found in the medium range for the difference between Internet use and educational status (Cramer’s V = 0.446) and relationship status (Cramer’s V = 0.302), as well as in the small range for social activities (Cramer’s V = 0.266). Table 4 shows health and psychological aspects according to online and offline users, as well as for the Italian total sample. Significant differences (*p* < 0.001) between online and offline users are found in terms of happiness, life satisfaction, trust in other people, subjective health, and participation in social activities. Effect sizes range from small to medium, with age showing the largest effect size at *r* = 0.430 and life satisfaction at *r* = 0.198.

With regard to religiousness, an average value of 5.66 (±1.79) was calculated for online users. Offliners showed a mean value of 7.10 (±2.38). The difference between the two groups is significant in the Mann–Whitney U Test (*p* ≤ 0.001). The Italian overall sample shows a religiousness of 6.54 (±2.60), with five missing values.

#### 3.1.2. France

Overall, the average age of the persons included in France is 74.46 (±6.98) years. The average number of children is about 2 ($\bar{X}$ = 2.37 ± 1.16), and the average number of grandchildren is more than 3 ($\bar{X}$ = 3.62 ± 2.85). Onliners are, on average, 72.72 (±6.16) years old and have, on average, 2 children ($\bar{X}$ = 2.19 ± 0.90) and slightly more than 3 grandchildren ($\bar{X}$ = 3.22 ± 2.40). In contrast, offliners are older on average at 77.05 (±7.34) years, have more children ($\bar{X}$ = 2.65 ± 1.45), and have, on average, 4 grandchildren ($\bar{X}$ = 4.27 ± 3.36). There were no missing values with respect to the age variable. However, there were missing values for the number of children (n = 129) and grandchildren (n = 147).

Table 5 shows the sociodemographic characteristics broken down by Internet use and for the French sample as a whole. It shows that aspects of low social status, such as female gender and low educational status, are found significantly more often among offliners than onliners (*p* < 0.001). They are also less likely to be married or in a partnership, more likely to be divorced, and more likely to be single (*p* < 0.001).

The largest effect sizes in the chi-square test are found in the medium range for the difference between Internet use and educational status (Cramer’s V = 0.386) and relationship status (Cramer’s V = 0.324), as well as in the small range for social activities (Cramer’s V = 0.171). Table 6 shows health and psychological aspects according to online and offline users, as well as for the French total sample. Significant differences (*p* < 0.001) between online and offline users are found in terms of happiness, life satisfaction, trust in other people, subjective health, and participation in social activities. Effect sizes range from small to medium, with age showing the largest effect size at *r* = 0.286 and life satisfaction at *r* = 0.122.

With regard to religiousness, an average value of 5.02 (±3.34) was calculated for online users. Offliners showed a mean value of 6.11 (±3.24). The difference between the two groups is significant in the Mann–Whitney U Test (*p* ≤ 0.001). The French overall sample shows a religiousness of 5.45 (±3.34), with 27 missing values.

#### 3.1.3. Germany

Overall, the average age of the persons included in Germany was 73.91 (±6.66) years. The average number of children is about 2 ($\bar{X}$ = 2.13 ± 1.00), and the average number of grandchildren is just under 3 ($\bar{X}$ = 2.77 ± 2.52). Onliners are, on average, 71.82 (±5.36) years old and have, on average, just under 2 children ($\bar{X}$ = 1.99 ± 0.88) and more than 2 grandchildren ($\bar{X}$ = 2.63 ± 2.31). In contrast, offliners are slightly but significantly (*p* < 0.001) older on average at 76.65 (±7.18) years and have significantly (*p* > 0.001) more children ($\bar{X}$ = 2.63 ± 1.11) and, on average, just under 3 grandchildren ($\bar{X}$ = 2.94 ± 2.76). With regard to the number of grandchildren, no significant difference was found between onliners and offliners in the Mann–Whitney U Test (*p* = 0.241). There were no missing values with respect to the age variable. However, there were missing values for the number of children (n = 252) and grandchildren (n = 275).

Table 7 shows the sociodemographic characteristics broken down by Internet use and for the German sample as a whole. It shows that aspects of low social status, such as female gender, low educational status, and the presence of a migration background, are found significantly more often among offliners than onliners (*p* < 0.001). They are also less likely to be married or in a partnership, more likely to be divorced, and more likely to be single (*p* < 0.001).

The largest effect sizes in the chi-square test are found in the medium range for the difference between Internet use and educational status (Cramer’s V = 0.375) and relationship status (Cramer’s V = 0.335), as well as in the small range for social activities (Cramer’s V = 0.216). Table 8 shows health and psychological aspects according to online and offline users, as well as for the German total sample. Significant differences (*p* < 0.001) between online and offline users are found in terms of happiness, life satisfaction, trust in other people, subjective health, and participation in social activities. Effect sizes range from small to medium, with age showing the largest effect size at *r* = 0.339 and life satisfaction at *r* = 0.176.

With regard to religiousness, an average value of 4.60 (±3.04) was calculated for online users. Offliners showed a mean value of 4.75 (±2.91). The difference between the two groups is not significant in the Mann–Whitney U Test (*p* = 0.376). The German overall sample shows a religiousness of 4.66 (±2.98), with seven missing values.

### 3.2. Analysis of the Results from Japan Data

First, all persons were excluded from the data set who were younger than 65 years at the time of the survey and did not live in Japan (Table 9).

Pearson chi-square tests revealed that variables such as age, dating status, health status, average monthly income, life satisfaction, and social activities were dependent on the number of onliners or offliners in Japan. That is, onliners were more likely to be younger than 70, have a partner, be healthier, and have a higher monthly income. These individuals were also more likely to be involved in social activities outside of work.

## 4. Discussion

The analysis aimed to provide an overview of Internet usage among individuals aged 65 and older, identifying differences between those who use and those who do not use the Internet. The results serve as crucial input for the future implementation of the e-VITA coach, guiding considerations for groups that may need special attention in development, particularly those with no prior experience with digital technologies. To achieve this, data from the 2018 European Social Survey (ESS) was analyzed for Italy, France, and Germany, alongside Japanese data from the 9th International Comparative Survey on the Lives and Attitudes of the Elderly. The findings reveal that over half of German (56.96%) and French (59.9%) adults aged 65 and above are Internet users. However, in Italy, only 39.14% of respondents in this age group use the Internet. Another study [13] highlights that digitized older individuals in Italy, particularly those aged 65 to 74, form a minority, indicating a digital divide influenced by socio-economic dimensions. Possession and use of information and communication technology (ICT) correlate with better socioeconomic conditions, physical activity, larger social networks, lower perceptions of aging, general personal satisfaction, interests, and self-confidence.

In Japan, the internet usage rate is challenging to calculate precisely. A 2020 survey by the Ministry of Internal Affairs and Communications indicates a sharp decline in Internet usage as age increases: 82.7% for ages 60–69, 59.6% for ages 70–79, and 25.6% for ages 80 and older. The standardized data collection between countries allows for a direct comparison of results, although the dichotomization of Internet use receives criticism for oversimplifying the heterogeneity of seniors as a user group [14]. Socioeconomic differences between online and offline users reveal that online users in France, Germany, and Italy are more likely to have medium or high levels of education. For example, older adults who use the Internet to obtain health information show significantly better overall health and happiness than those who seek information offline only [15]. However, these specific uses of the Internet do not emerge from the ESS data. With regard to socioeconomic differences between online and offline users, online users in France, Germany, and Italy are significantly more likely to have a medium or high level of education. A study conducted by Quittschalle et al. [16] in Germany delved into the factors influencing Internet usage among individuals aged 75 and above. The research identified that male gender, lower age, and higher educational attainment were pivotal factors in increasing the likelihood of Internet use. Specifically, male gender and a higher level of education emerged as the strongest predictors. The authors attribute this trend to prevailing stereotypes, particularly among the “old” older adults, associating technology use more with the male role. However, they suggest that this circumstance might evolve with subsequent generations. Additionally, Hunsaker and Hargittai [1], in their review, refer to a study [17] indicating that men exhibit a significantly higher rate of self-confidence in Internet use compared to women. For the development and implementation of e-VITA, it is crucial to be mindful of potential gender stereotypes and actively encourage older women to embrace technology. While gender-specific effects may diminish with age, increased self-efficacy, and heightened interest in the Internet, studies present inconsistent findings on the relationship between gender and Internet use. Some studies suggest no differences, while others assert that women use the Internet more frequently than men [18]. Therefore, careful consideration and ongoing awareness of gender dynamics are imperative in ensuring the equitable engagement of all users, irrespective of gender, with e-VITA. Furthermore, marital status plays a significant role, with married or partnered individuals more likely to be online users in all three European countries. Although the definitions of onliner and offliner in Japan differ slightly from those in the EU survey, the outcomes are strikingly similar. The presence of a partner or family member encouraging Internet use could contribute to this trend [19]. However, contradictory findings indicate that offline users more often have more children and grandchildren, emphasizing the need for further investigation into the influence of family dynamics on older adults’ Internet use. This phenomenon should be investigated in further studies using inferential statistical methods in order to shed more light on the influence of (grand)children on the use of the Internet by older adults. The analysis also notes that among offline users, a higher percentage had an immigrant background in Germany, indicating a potential language barrier [19]. Another study by Samkange-Zeeb et al. [20] investigated in eight culturally diverse neighborhoods of four different countries (two each in Birmingham, UK; Bremen, Germany; Lisbon, Portugal; and Uppsala, Sweden) what influence a migration background has on the use of Internet-based health information. The study shows that first-generation migrants, poor knowledge of the native language, higher age and lower education level are associated with lower odds regarding the use of Internet-based health information. The researchers attribute this to existing language barriers within countries, poorer living conditions, and limited access to information. Addressing the needs of older migrants in the development of e-VITA is crucial, and considerations such as translation into languages other than the national language may be relevant. At a later stage, it may be worthwhile to explore the translation of the coaching materials into languages other than the national language or languages known by the coach. Additionally, considering the bilingualism of the coach could be beneficial. Furthermore, Samakange-Zeeb et al. [20] identified varying effects across countries. Notably, the age effect disappeared entirely in the analysis conducted in Uppsala, Sweden. This underscores the significance of acknowledging cultural and structural differences between countries, a consideration integral to the e-VITA project. In all European countries, this analysis found that online users experience significantly higher health satisfaction, increased life satisfaction, and greater overall happiness. Consistent with prior research, Internet use among older individuals is linked to better self-reported health and a reduced risk of functional limitations [1,21,22,23]. Notably, Internet use itself plays a minor role, with other health or social factors being more influential. The analysis also highlights a low effect size between Internet use and health. Comparing activities with peers of the same age, offliners are notably more likely to report engaging in fewer social activities. Szabo et al. longitudinal study [24] supports this, indicating that Internet use can enhance social engagement and reduce loneliness. However, the researchers emphasize that merely being online is not sufficient; how individuals engage with the digital space matters. Therefore, social use of the Internet, such as contacting friends or family, emerges as the sole predictor of reducing loneliness. These results underscore the relevance of supporting older individuals in using the Internet for social interactions. e-VITA offers a first basis for this with its planned integrated social platform.

Furthermore, Szabo et al. [24] show that Internet use for social, information-related (e.g., reading health information), or instrumental intentions (e.g., online banking) can improve overall well-being over time, as it is associated with reduced loneliness and/or increased social engagement. However, it must be taken into account at this point that the determination of social activities by means of the question “*Compared to other people of your age, how often would you say you take part in social activities?*” can be accompanied by a certain bias. It is conceivable that the perceptions of the activities of “a person of the same age” can differ significantly, so that the respondents may over- or underestimate their social activities. As for the social activities of older people in Japan, the percentage of onliners who are employed is much higher than that of offliners (41.1% vs. 15.9%). This may be due to the fact that they often use the Internet for work. In terms of religiousness, offliners consider themselves to be more religious than onliners, but this difference in the Mann–Whitney U test is only significant in France and Italy. Considering this result, integrating spirituality and religiosity into the e-VITA Coach could serve as a starting point, particularly in these countries, to encourage individuals unfamiliar with modern technologies to engage, given their potential support for religious practices. However, a potential obstacle to implementing the e-VITA Coach arises from the present analysis, indicating that offliners generally exhibit lower trust in others compared to onliners. This underscores the importance of transparently presenting all functions, potential benefits, and risks to users, allowing them time to explore and become familiar with the coach. In Japan, the analysis reveals that a small proportion of older individuals, less than 5%, are religiously or politically active, irrespective of online or offline status. This suggests a limited necessity to incorporate religious or political components into e-VITA coaching in Japan.

## 5. Conclusions

The paper provides an initial examination of the environment and viewpoint of elderly end-users, aiming to delineate successful strategies and prospects for introducing an active and healthy aging coaching system into the market and society. A subsequent round of analysis is in the pipeline, with the goal of acquiring more precise information tailored to the system’s diffusion, accounting for its distinctive features. Scrutinizing digital behavior remains an indispensable aspect of any user-centered design approach, ensuring the system’s embrace post-deployment.

The present study has limitations, even if interesting conclusions can be derived from the analysis of survey data. It is important to consider more precise questions, and indicators should be analyzed. For example, attitudes toward technology itself and attitudes toward the relative technologies need to be explored to understand if there are factors underlying the importance given to health status. These factors can act as drivers for ensuring a massive use of technologies and facilitating the integration of everyday and healthcare technology into the lives of older people.

It is also interesting to examine the relationship between technology mediators, such as family members, doctors, neighbors, or friends, and the influence of social networks on the decision to use technology. The awareness of technological advice can be easily found and retrieved by older users.

Finally, the data suggest the importance of dedicated training for using technology as a driver and motivator for the adoption of technologies, even in the case of non-offline users who are already familiar or comfortable with using technological or online tools.

## Figures and Tables

**Table 1:** Operationalization of the socioeconomic variables.

Variable	Collection in ESS	Inclusion in the Analysis
Age	Calculated from the indication of the date of birth	As defined in the ESS
Number of children	Collected from the information provided in the question about the “Number of Children ever given birth to/fathered”	As defined in the ESS
Number of Grandchildren	Collected from the information given when asked about the number of grandchildren	As defined in the ESS
Gender	Collected from the question about whether a person is male or female;	As defined in the ESS
Relationship Status	Collected from the question “You just told me that you live with your husband/wife/partner. Which one of the descriptions on this card describes your relationship to them?” (Legally married; In a legally registered civil union; Living with my partner—not legally recognized; Living with my partner—legally recognized; Legally separated; Legally divorced/Civil union dissolved) and the question “This question is about your legal marital status not about who you may or may not be living *with. Which one of the descriptions on this card describes your legal marital status now?*” (Legally married; In a legally registered civil union; legally separated; Legally divorced/Civil union dissolved; Widowed/Civil partner died; None of these (NEVER married or in a legally registered civil union))	Coded in one variable: Married/Partnership (includes registered civil unions); Divorced/Separated (includes dissolved registered civil unions); Widowed, Single (never married or in a legally registered civil union)
Level of Education	Classification of educational attainment using the ES-ISCED generated variable in the ESS	Reduction to 3 categories: Low = ES-ISCED I and II Medium = ES-ISCED IIIb, IIIa and IV High = ES-ISCED V1 and V2
Migration background	Not directly collected in the ESS	Calculated from whether respondents whose mother or father were born in the country of destination. A migration background was assumed if the respondent, his/her mother, or father were not born in the country of destination (Destatis, n.d.)

Variable

Collection in ESS

Inclusion in the Analysis

**Table 2:** Operationalization of the health-related and psychological variables.

Variable	Collection in ESS	Inclusion in the Analysis
Happiness	Collected from the question “*taking all things together, how happy would you say you are?*” (0 extremely unhappy to 10 extremely happy)	As defined in the ESS
Satisfaction with life	Collected from “*All things considered, how satisfied are you with your life as a whole nowadays? Please answer using this card, where 0 means extremely dissatisfied and 10 means extremely satisfied.*”	As defined in the ESS
Most People can be trusted	Collected from the question “*generally speaking, would you say that most people can be trusted, or that you can’t be too careful in dealing with people? Please tell me on a score of 0 to 10, where 0 means you can’t be too careful and 10 means that most people can be trusted.*” (0 You can’t be too careful to 10 Most people can be trusted)	As defined in the ESS
Satisfaction with Health	Collected from the question “*how is your health in general? Would you say it is...*” (1 Very Good to 5 Very Bad)	Dichotomized, Rather good = 1–3 Rather bad = 4–5
Take part in social activities compared to others of same age	Compared to other people of your age, how often would you say you take part in social activities? (1 Much less than most to 5 Much more than most)	Dichotomized, Less = 1–2 Same or more = 3–5

**Table 3:** Socioeconomic characteristics by internet use and total, Italy (column%).

		Internet Use, Dichotomized		
		Onliner (%)	Offliner (%)	Total (%)
Gender *	Male	50.56	38.31	43.11
	Female	49.44	61.69	56.89
	Missing value (n = 0)			
Relationship Status *	Married/Partnership	70.72	58.57	63.32
	Divorced/Separated	9.97	1.59	4.86
	Widowed	12.42	35.43	26.45
	Single	6.88	4.41	5.37
	Missing values (n = 15)			
Level of education, categorized *	Low	47.87	87.13	71.84
	Mid	34.74	11.76	20.71
	High	17.38	1.11	7.45
	Missing values (n = 6)			
**Migration background**	Yes	3.11	2.41	2.68
	No	96.89	97.59	97.32
	Missing values (n = 2)			

* Pearson chi-square (*p* < 0.001).

**Table 4:** Health-related and psychological characteristics by Internet use and total, Italy (column%, mean, and SD).

		Internet Use, Dichotomized					
		Onliner		Offliner		Total	
		Mean (sd)	%	Mean (sd)	%	Mean (sd)	%
**How happy are you** **Missing values (n = 1)**		7.02 (1.83)	-	6.18 (2.09)	-	6.51 (2.03)	-

**How satisfied with life as a whole** **Missing values (n = 5)**		7.17 (1.71)	-	6.26 (2.27)	-	6.62 (2.12)	-
**Most people can be trusted** **Missing values (n = 3)**		5.11 (2.31)	-	4.05 (2.44)	-	4.46 (2.44)	-
Satisfaction with health *	Rather good	-	91.38	-	78.46	-	83.53
	Rather bad	-	8.62	-	21.54	-	16.47
	Missing values (n = 4)						
Take part in social activities *	Less	-	19.14	-	45.00	-	34.72
	Same or more	-	80.86	-	55.00	-	65.28
	Missing values (n = 6)						

* Pearson chi-square (*p* < 0.001).

**Table 5:** Socioeconomic characteristics by internet use and total, France (column%).

		Internet Use, Dichotomized		
		Onliner (%)	Offliner (%)	Total (%)
Gender *	Male	50.90	35.82	44.85
	Female	49.10	64.18	55.15
	Missing value (n = 0)			
Relationship Status *	Married/Partnership	71.21	45.69	60.96
	Divorced/Separated	13.04	10.26	11.92
	Widowed	11.31	37.49	21.82
	Single	4.44	6.56	5.30
	Missing values (n = 5)			
Level of education, categorized *	Low	36.50	74.21	51.56
	Mid	46.93	23.90	37.73
	High	16.57	1.88	10.71
	Missing values (n = 4)			
**Migration background**	Yes	21.56	20.04	20.95
	No	78.44	79.96	79.05
	Missing values (n = 12)			

* Pearson chi-square (*p* < 0.001).

**Table 6:** Health-related and psychological characteristics by Internet use and total, France (column%, mean, and SD).

		Internet Use, Dichotomized					
		Onliner		Offliner		Total	
		Mean (sd)	%	Mean (sd)	%	Mean (sd)	%
**How happy are you** **Missing values (n = 7)**		7.33 (1.56)	-	6.92 (1.97)	-	7.17 (1.75)	-

**How satisfied with life as a whole** **Missing values (n = 4)**		6.65 (2.04)	-	6.02 (2.50)	-	6.40 (2.25)	-
**Most people can be trusted** **Missing values (n = 1)**		4.68 (1.93)	-	4.00 (2.48)	-	4.41 (2.19)	-
Satisfaction with health *	Rather good	-	91.21	-	81.54	-	87.32
	Rather bad	-	8.79	-	18.46	-	12.68
	Missing values (n = 4)						
Take part in social activities *	Less	-	17.27	-	31.97	-	23.16
	Same or more	-	82.73	-	68.03	-	76.84
	Missing values (n = 23)						

* Pearson chi-square (*p* < 0.001).

**Table 7:** Socioeconomic characteristics by internet use and total, Germany (column%).

		Internet Use, Dichotomized		
		Onliner (%)	Offliner (%)	Total (%)
Gender *	Male	51.68	41.21	47.14
	Female	48.32	58.79	52.86
	Missing value (n = 0)			
Relationship Status *	Married/Partnership	78.37	47.83	65.16
	Divorced/Separated	7.23	9.58	8.25
	Widowed	11.06	36.28	21.97
	Single	3.34	6.30	4.62
	Missing values (n = 7)			
Level of education, categorized *	Low	12.33	41.33	24.82
	Mid	67.21	54.72	61.83
	High	20.46	3.94	13.35
	Missing values (n = 7)			
Migration background *	Yes	11.61	20.42	15.36
	No	88.39	79.58	84.64
	Missing values (n = 26)			

* Pearson chi-square (*p* < 0.001).

**Table 8:** Health-related and psychological characteristics by Internet use and total, Germany (column%, Mean, and SD).

		Internet Use, Dichotomized					
		Onliner		Offliner		Total	
		Mean (sd)	%	Mean (sd)	%	Mean (sd)	%
**How happy are you** **Missing values (n = 0)**		8.07 (1.59)	-	7.48 (2.22)	-	7.81 (1.91)	-

**How satisfied with life as a whole** **Missing values (n = 0)**		8.16 (1.73)	-	7.39 (2.18)	-	7.83 (1.97)	-
**Most people can be trusted** **Missing values (n = 0)**		5.66 (2.28)	-	4.98 (2.70)	-	5.37 (2.49)	-
Satisfaction with health *	Rather good	-	89.99	-	78.80	-	85.14
	Rather bad	-	10.01	-	21.20	-	14.86
	Missing values (n = 3)						
Take part in social activities *	Less	-	30.15	-	51.38	-	39.31
	Same or more	-	69.85	-	48.62	-	60.69
	Missing values (n = 8)						

* Pearson chi-square (*p* < 0.001).

**Table 9:** Socioeconomic characteristics by internet use and total, Japan.

		Internet Use, Dichotomized		
		Onliner (%)	Offliner (%)	Total (%)
Gender *	Male	49.14	38.80	47.66
	Female	50.86	61.20	52.34
Age *	60–64	16.11	6.56	14.74
	65–69	25.20	7.65	22.70
	70–74	28.03	22.95	27.30
	75–79	17.47	17.49	17.47
	80+	13.19	45.36	17.78
Relationship Status **	Married/Partnership	74.34	47.54	70.51
	Divorced/Separated	6.19	6.01	6.16
	Widowed	12.92	32.79	15.76
	Single	4.82	9.84	5.54
	Single for health or care reasons	1.36	3.28	1.64
	Missing values (n = 9)	0.36	0.55	0.39
Health Status **	Healthy	54.68	33.33	51.64
	Not very healthy, but not sick	38.76	51.91	40.64
	I am sick and sometimes in bed	3.91	9.84	4.76
	I am sick and stay in bed all day	0.45	3.83	0.94
	Missing values (n = 33)	2.18	1.09	2.03
Average monthly income (total of husband and wife) **	Less than 50,000 yen	1.09	7.65	2.03
	50,000 to 99,999 yen	7.92	19.67	9.59
	100,000 to 199,999 yen	24.57	36.61	26.29
	200,000 to 299,999 yen	30.57	19.67	29.02
	300 to 399,999 yen	15.92	7.65	14.74
	400,000 to 499,999 yen	7.64	1.64	6.79
	500,000 to 599,999 yen	3.28	0.55	2.89
	600,000 yen–	5.10	0.00	4.37
	No income	0.27	0.00	0.23
	Missing values (n = 60)	3.64	6.56	4.06
Life Satisfaction *	Satisfied	83.44	73.22	81.98
	Dissatisfied	14.83	24.04	16.15
	Missing values (n = 31)	1.73	2.73	1.87
**Take part in social activities**	Work with income **	41.13	15.85	37.52
	Other social activities **	43.59	24.04	40.80
	(Religious and political activities) *	4.37	1.64	3.12

Pearson chi-square (* *p* < 0.01, ** *p* < 0.001).

## Data Availability

The data presented in this study are available in the article itself.
